# Editorial of the Special Issue Antimicrobial Polymers

**DOI:** 10.3390/ijms140918002

**Published:** 2013-09-03

**Authors:** Antonella Piozzi, Iolanda Francolini

**Affiliations:** Department of Chemistry, Sapienza University of Rome, P.le Aldo Moro 5, 00185 Rome, Italy; E-Mail: iolanda.francolini@uniroma1.it

**Keywords:** antimicrobial polymers, antifouling polymers, antimicrobial agent delivery systems, cationic polymers, microbial biofilms, titania, silver, magnetic nanoparticles, nanocomposites, chitosan, usnic acid

## Abstract

The special issue “Antimicrobial Polymers” includes research and review papers concerning the recent advances on preparation of antimicrobial polymers and their relevance to industrial settings and biomedical field. Antimicrobial polymers have recently emerged as promising candidates to fight microbial contamination onto surfaces thanks to their interesting properties. In this special issue, the main strategies pursued for developing antimicrobial polymers, including polymer impregnation with antimicrobial agents or synthesis of polymers bearing antimicrobial moieties, were discussed. The future application of these polymers either in industrial or healthcare settings could result in an extremely positive impact not only at the economic level but also for the improvement of quality of life.

## 1. Communication

Microbial contamination is of great concern in many area including medical devices, health care products, textiles, water purification systems, food packaging and storage. Any material, either synthetic or natural, can be, in fact, easily colonized by microorganisms that then proliferate on its surface building up a peculiar tridimensional structure known as a biofilm.

Biofilms can cause significant damage to industrial settings with consequent economic losses. For example, in industrial processes for water and wastewater treatment, biofilm formation on filtration membranes can greatly compromise the efficiency of treatment processes. In drinking water distribution systems, microbial colonization of the pipe walls can decrease water quality by increasing bacteria level, reducing the dissolved oxygen and changing water taste and odor.

Microbial contamination of food products during storage, processing or distribution is an important hygienic and health problem since it can cause food spoilage and food-borne illnesses.

In the biomedical field, biofilm formation of the surfaces of implantable medical devices, such as intravascular catheters, urinary catheters and orthopedic implants, is associated with the development of antibiotic-resistant, often life-threatening, infections.

Biofilm eradication is considered a difficult task, since the biofilm-like mode of growth permits the survival of microorganisms under high levels of antibiotic and extreme conditions of temperature, pH, ionic strength and shear stress. Therefore, prevention of biofilm formation represents a great challenge for both industry and research.

The present special issue includes research and review papers on recent advances obtained in the development of antimicrobial polymers and their relevance to industrial and healthcare settings. Antimicrobial polymers have recently emerged as promising candidates to fight microbial contamination onto surfaces thanks to their interesting properties. In fact, the versatility of the macromolecular chemistry facilitates the tailoring of polymer physico-chemical properties (molecular weight, composition, functionality) to fulfill different applications. Polymers have become the materials of choice in numerous industrial settings and particularly in the biomedical field, where they are used for a wide range of products among which implantable medical devices are the prevailing ones.

Two main strategies can be pursued to develop antimicrobial polymers: (i) polymer impregnation with antibiotics or other biocides; (ii) polymer functionalization with groups exerting antimicrobial activity.

In the first approach, the polymer acts as a carrier for one or more antimicrobial agent that once released from the polymer exerts its action (antimicrobial agent-releasing polymers). In the second approach, bactericidal functionalities, such as quaternary amine containing compounds or phosphonium salts, are introduced in the polymer to obtain intrinsically antimicrobial polymers (biocidal polymers). Biocidal polymers do not release antimicrobial substances but exert their killing action when microorganisms contact the surface.

To obtain antimicrobial agent-releasing polymers, anionic or cationic surfactants, antibiotics, antiseptics or heavy metals can be applied to the polymer by: (a) physical adsorption; (b) impregnation, (c) complexation or (d) conjugation via hydrolytically labile linkages. Particularly, drug impregnation is the most pursued method since it is easy to perform and can permit the loading of significant drug amounts by increasing polymer matrix porosity.

Li and coworkers [1] developed antimicrobial polymer coatings for industrial applications by loading sodium dodecyl sulfate (SDS) into nanoporous polymers derived from self-assembled polybutadiene-*b*-polydimethylsiloxane block copolymer. Some attractive features of this system are the possibility to tune pore size, to have narrow pore size distribution, and to adjust its chemical and mechanical properties. The obtained SDS-loaded nanoporous polymer films were shown to be more active in preventing bacterial adhesion and reducing biofilm formation when challenged with the gram-negative *Escherichia coli* than the gram-positive *Staphylococcus epidermidis*.

Different amounts of TiO_2_ nanoparticles were impregnated into a biodegradable polycaprolactone (PCL) matrix by Munoz-Bonilla *et al.* to obtain a bioactive food packaging [2]. TiO_2_ nanoparticles were well dispersed into the PCL matrix and did not significantly change PCL crystalline structure, phase transitions and thermal stability. Since TiO_2_ is a light-induced biocidal substance, antibacterial tests were performed by irradiating samples with light at 280 (UV) and 500 (visible) nm for different time periods. Better results were obtained by UV irradiation where a log-reduction ranging from 3 to 5.6, depending on TiO_2_ content, was observed against *E. coli* and from 4 to 5 for *Staphylococcus aureus*.

Silver has always attracted much attention for its broad-spectrum antibacterial activity and has become among the most employed antimicrobial agents for impregnation in biomedical applications. Different approaches have been investigated to incorporate silver in polymeric substrates. A conventional approach is by the deposition of metallic silver directly onto the surface of the substrate, for example by vapour coating, sputter coating, or ion beam coating. An approach based on impregnation of materials with silver nanoparticles (Ag-NPs) rather than coating has been more recently developed.

Several AgNPs-containing systems have been prepared to promote wound healing [3], to hinder dental plaque formation [4] or colonization of denture soft lining materials [5].

Nanogels, cross-linked hydrophilic polymers, have many practical applications including drug delivery systems and as filler materials in the coating industry. Different physical and chemical methods are used for polymer crosslinking. Especially in the case of chemical crosslinking, some drawbacks, such as toxicity and elimination of crosslinker after reaction, can be found.

To simultaneously obtain sterilized and crosslinked materials, Choi *et al.* have used radiation technology for preparation of polyacrylic acid-based nanogels containing silver nanoparticles (Ag/PAAc) [3]. The reduction of Ag^+^ ions without using reducing agents was easily obtained as shown by FE-SEM-EDX analysis. Particle size analysis showed that the average size of the Ag/PAAc nanogels were approximately 200 nm at 100 kGy. Also, it was verified that while the size of PAAc nanogels decreased with increasing electron beam irradiation doses, the zeta potential value increased demonstrating a poor material aggregation. The obtained Ag/PAAc nanogels exhibited good antibacterial activity against both Gram-negative and Gram-positive bacteria and by *in vivo* experiments a good healing effect of the Ag/PAAc nanogel pastes was observed.

It is generally recognized that metallic-based nanoparticles are stabilized by a biocompatible polymer coating. Chitosan (CS), polysaccharide derived from naturally occurring chitin, is widely investigated for many applications thanks to its biocomopatibility, interesting biological properties and its versatility to be prepared into different forms such as nanoparticles, fibers and film.

Di Giulio *et al.* developed a nanocomposite system based on a lactose-substituted chitosan and silver nanoparticles (Chitlac-nAg) [4] for hindering dental plaque formation. The Chitlac-nAg system, at very low nAg concentration (0.1% *v*/*v*), provided the inhibition of the growth of *Streptococccus mutans* and *Streptococcus oralis* planktonic phase, except for *Streptococcus mitis* that was instead inhibited at one step less. On the contrary, on preformed biofilm, Chitlac-nAg was able to inhibit the bacterial growth at a value of 0.2% on the supernatant phase and on the mature biofilm. For *S. mitis* the Chitlac-nAg inhibitory concentration on biofilm was 0.1%. The silver nanoparticle polysaccharide system showed a good antimicrobial behavior also when studied against saliva.

To reduce the colonization of denture soft lining materials, silver nanoparticles ranging from 0 to 200 ppm were incorporated into composites by Chladek *et al.* [5]. The effect of antimicrobial agent incorporation on sorption, solubility, hardness and tensile bond strength of the composites was investigated. It was verified that an increase in the silver concentration caused a decrease in hardness, in bond strength and an increase in sorption and solubility as well as a change in the failure type of the samples. A good *in vitro* antifungal efficiency and physical properties no worse than those of the material without silver were found for composites with concentrations of silver nanoparticles of up to 40 ppm.

To prevent infections, silver, iodine, polyhexamethylene biguanide (PHMB) or chlorhexidine are the most commonly used antimicrobial agents in wound dressings. Infected wounds can have serious local and systemic complications. While an important local complication of infected wounds is a non-healing wound, the infection of the surrounding tissues may cause a skin infection or an acute or chronic bone infection. If bacteria get into bloodstream, they may instead cause infection in other areas of the body.

Forstner *et al.* [6] have investigated the antimicrobial efficacy and the influence on bacterial growth kinetics of a flexible methacrylate powder dressing (Altrazeal^®^) loaded with different combinations of cationic and anionic antiseptics, specifically PHMB, Povidone-iodine (PVP-iodine), Octenidine-dihydrochloride (OCT), Betaine and Phenoxyethanol. Altrazeal^®^ is based on dehydrated hydrogel modified into small particles containing a poly-2-hydroxyethyl-/poly-2-hydroxypropyl (pHEMA/pHPMA)-methacrylate backbone and terminal hydroxyl group. *In vitro* model of porcine wound, contaminated with *S. aureus*, evidenced that an individually shaped and controlled antibacterial effect may be obtained using the same type of wound dressing but different combinations of cationic and anionic antiseptics. In particular, the highest bacterial reduction was found for PVP-iodine at 24 h followed by OCT at 48 h. Altrazeal^®^ loaded with PHMB alone showed a stable bacterial load up to 120 h while by using the combination PHMB and Betaine, a bacterial growth inhibition during the first 48 h and a slightly increase of bacterial number at 120 h was observed.

The development of biocidal polymers by introduction of antimicrobial functional groups is proving to be an alternative to the use of low molecular weight antimicrobials. Specially, polymers containing positively charged groups such as phosphonium, biguanide or quaternary ammonium groups have likelihood to succeed in preventing infections and combating multi-drug bacterial resistance.

Carmona-Ribeiro and Dias de Melo Carrasco have extensively discussed recent research on macromolecular antimicrobial agents such as cationic polymers, peptoids, peptides and lipids [7]. In this review, the explored approaches to enhance polymer antimicrobial activity or to deliver bioactive molecules were also reported. These approaches involve the formation of molecular or supramolecular structures by grafting or self-assembling of antimicrobial polymers to inert or non inert materials.

Chitosan has been widely used in agriculture, in medicine and in industrial applications as a macromolecular antimicrobial agent. To improve CS antimicrobial properties and its solubility in water over a large range of pH values, different chemical modifications aimed at obtaining CS derivatives have been investigated. Tan *et al.*, in their review [8], have attempted to summarize the current findings on the antimicrobial properties of quaternized CS synthesized using different methods. In addition, the potential biomedical applications of quaternized CS in orthopedics as well as the mechanism of its antimicrobial action have been discussed.

Generally, the interaction between an antibacterial cationic agent and the bacterial cell wall leads to disruption of lipid bilayer and then to an enhancement of membrane permeability. Horvath *et al.* have investigated the interaction between an antibacterial agent and the lipid bilayer by using a surface sensitive optical waveguide spectroscopy [9]. Specifically, the authors used branched poly(ethylene imine) (PEI) functionalized with quaternary ammonium groups, polyethylene oxide (PEO) and alkyl chains as an antibacterial agent and 1-palmitoyl-2-oleoyl-sn-glycero-3-phosphocholine (POPC) as a material to prepare unilamellar lipid vesicles. These latter after adsorption and spreading on the waveguide’s surface formed the solid supported lipid bilayer. In addition, to better reproduce the condition resembles to contact bacteria-antibacterial coating, the polymer was continuously introduced in the flow cell of the optical waveguide lightmode spectrometer (OWLS). Measurements showed that the compact lipid bilayer had positive birefringence while the optical birefringence of antibacterial polyelectrolyte layer was negative. From these experimental findings, the authors developed a composite layer model to deduce the birefringence of the surface layer during the interaction between the lipid bilayer and the polyelectrolyte. This study demonstrated that this technique can be useful to understand the mechanism of antibacterial effects leading to the disintegration of cell membranes without using additional labeling.

Fluorescent labeling is an important technique playing a key role in biological imaging and biological monitoring. Quantum dots (QDs) are a novel class of inorganic fluorophores which are gaining wide recognition as a result of their unique optical and chemical properties.

Xiao *et al.* obtained multifunctional hybrid microspheres with magnetic, antibacterial and fluorescent function by using poly(glycidyl methacrylate) (PGMA) as a cladding material [10]. In particular, magnetic microspheres were prepared from iron oxides and PGMA functionalized with ethanediamine (NH_2_-PGMA). After further surface functionalization with poly(hexamethylene guanidine hydrochloride) (PHGH), the microspheres exhibited excellent antibacterial activity against both Gram-positive and Gram-negative bacteria. Finally, the authors provided the hybrid microspheres with targeted localization and biological monitoring functions by the quantum dot CdTe introduction.

Radiology is another monitoring technique widely utilized to evaluate dental restoration and long-term stability of dental implant. Therefore radiopacity property of dental materials is very important. An adequate radiopacity allows dentists distinguishing existing restorations and primary caries, evaluating margins, overhangs and major voids and identifying any recurrent caries.

In order to endow a dental material with antibacterial and radio-opaque functions, He *et al.* [11] have incorporated a polymerizable antibacterial agent (2-dimethyl-2-dodecyl-1-methacryloxyethyl ammonium iodine, DDMAI) into 2,2-bis[4-(2-hydroxy-3-methacryloxypropyl)-phenyl]propane (Bis-GMA)/methyl methacrylate (MMA) dental resin system. The experimental findings evidenced that the dental resin with DDMAI had better antibacterial activity and radiopacity than that without methacrylate monomer. However, since DDMAI incorporation into Bis-GMA/MMA resin could reduce mechanical properties and increase water sorption and solubility, further optimization of resin formulation is needed for preclinical application.

The main advantage of using antimicrobial agent-releasing systems is the drug release targeted to the infection site. However, most of these systems have a short persistence of antimicrobial action, due to rather quick diffusion controlled release. In addition, the low systemic drug concentration reached after few days of drug release can increase the risk for selecting of antimicrobial resistant strains.

Fungal infection has become a leading cause of nosocomial infections. The *Candida* species are the most common fungi isolated from device-related infections, and biofilms formed by these fungal organisms are especially associated with drastically enhanced resistance against many antimicrobial agents. This resistance contributes to the persistence of the infection despite antifungal therapy.

Antimicrobial photodynamic inactivation (PDI) has been developed as an alternative therapeutic treatment against bacterial and fungal local infections. This treatment is particularly utilized in case of drug-resistant strains. Being *Candida albicans* less susceptible to PDI, high doses of photosensitizer and light energy are needed to treat infectious diseases related to this strain.

To potentiate PDI efficacy against *C. albicans*, Chien *et al.* [12] studied the combination of PDI and chitosan against a wild-type strain of *C. albicans* and three fluconazole-resistant clinical isolates, in planktonic and biofilm forms. The results showed that chitosan, after cell damage by PDI, was able to enhance the effect of PDI mediated by toluidine blue O. In addition, a reduction in the PDI treatment conditions needed to completely eradicate *C. albicans* was possible with increasing chitosan concentration or incubation time.

Another valid approach developed to prevent the emergence of resistant pathogens involves the use of proper antimicrobial combinations having different mechanisms action. Particularly, advantages in the employment of an intrinsically antimicrobial polymer as a drug carrier, rather than of an inert one, have been exploited by Francolini *et al.* [13] to reduce the development of drug resistant bacteria. The authors complexed usnic acid, a potent antimicrobial and anticancer agent, poorly soluble in water, to novel antimicrobial cationic polyacrylamides. The polymer/drug complexes promptly dissolved in water and possessed a greater antimicrobial activity against *Staphylococcus epidermidis* than both the free drug and the polymer alone. The best results were obtained with the complex based on the lowest molecular weight polymer and containing a low drug content. This improved killing effect was attributed to the reduced size of the complex that allowed its efficient cellular uptake.

## 2. Conclusions

In this special issue the promising performance of antimicrobial polymers in fighting microbial contamination onto surfaces and reducing multi-drug bacterial resistance were highlighted. We believe the future application of these polymers either in industrial or healthcare settings could result in an extremely positive impact not only at the economic level but also for the improvement of quality of life.
